# Hepatoprotective effect of β-myrcene pretreatment against acetaminophen-induced liver injury

**DOI:** 10.22038/AJP.2022.19493

**Published:** 2022

**Authors:** Gabriel Fernando Esteves Cardia, Francielli Maria de Souza Silva-Comar, Carla Indianara Bonetti, Edvalkia Magna Teobaldo da Rocha, Mayara Zagoto, Valeria do Amaral, Livia Bracht, Saulo Euclides Silva-Filho, Ciomar Aparecida Bersani-Amado, Roberto Kenji Nakamura Cuman

**Affiliations:** 1 *Department of Pharmacology and Therapeutics, State University of Maringá, Maringá, PR, Brazil*; 2 *Department of Biochemistry, State University of Maringá, Maringá, PR, Brazil*; 3 *Pharmaceutical Sciences, Food and Nutrition College, Federal University of Mato Grosso do Sul, Campo Grande, MS, Brazil*

**Keywords:** β-myrcene, Antioxidants, Acute hepatic failure, Liver diseases

## Abstract

**Objective:**

In the present study, the hepatoprotective effects of β-myrcene (MYR) on acetaminophen-induced hepatotoxicity were investigated.

**Materials and Methods:**

A total of 40 Balb/c mice were randomly divided into five groups as follows: 1) Normal control group which received only carboxymethylcellulose (CMC), the vehicle used to dissolve acetaminophen (N-acetyl-p-aminophenol, APAP, paracetamol) and MYR; 2) APAP group which received a single dose of acetaminophen (250 mg/kg) orally on day 7; 3) Silymarin group which received 200 mg/kg/day of silymarin; and 4 and 5) pretreatment groups in which, mice were treated with 100 or 200 mg/kg/day of MYR. Liver and blood samples were collected to analyze serum aminotransferases, inflammatory response, oxidative stress markers, and histopathological insults.

**Results:**

Our results showed that MYR pretreatment attenuated liver damage and restored liver cells function and integrity as it decreased the leakage of serum aminotransferases (alanine and aspartate aminotransferases (ALT and AST, respectively)) into the blood (p<0.01). MYR treatment also reduced levels of myeloperoxidase (MPO) activity and nitric oxide (NO) (p<0.001). In addition, MYR pretreatment demonstrated significant antioxidant activity by decreasing malondialdehyde (MDA), reactive oxygen species (ROS), and reduced glutathione (GSH) levels (p<0.001). Furthermore, it restored the hepatic level of superoxide dismutase (SOD), catalase (CAT), and oxidized glutathione (GSSG) (p<0.001).

**Conclusion:**

For the first time, our results showed that MYR treatment significantly improved liver function by reducing oxidative stress and the inflammatory response induced by APAP.

## Introduction

Liver diseases are responsible for millions of deaths each year, and one of the leading causes of liver damage is drug-induced hepatotoxicity due to its unpredictability and potentially fatal nature (Asrani et al., 2019 ▶). Acetaminophen (N-acetyl-p-aminophenol, APAP, paracetamol) is a clinically important drug that has been associated with liver injury. APAP is one of the most recommended and consumed analgesic and antipyretic in the world (Ghanem et al., 2016 ▶; Subramanya et al., 2018 ▶). Although its efficacy was confirmed at therapeutic dosages, APAP overdose can cause hepatotoxicity, promoting acute liver failure (ALF), and induce kidney damage in humans and animals (Athersuch et al., 2018 ▶; Chaudhuri et al., 2011 ▶; Saleem and Iftikhar, 2019 ▶). APAP intentional/unintentional intoxication remains the most common cause of ALF in Asian, American, and European countries. It is responsible for more cases of ALF than all other etiologies combined (Bernal et al., 2010 ▶; Ghanem et al., 2016 ▶). Besides, in many cases, liver transplantation is the only treatment option for the patient to survive (Tezcan et al., 2018 ▶). Therefore, there is a clinical need for effective treatments to reduce or reverse acute hepatotoxicity. It is important to highlight that APAP, at the therapeutic dose, is metabolized mainly by glucuronidation and sulfation to nontoxic metabolites and then, excreted. In contrast, an overdose of APAP (more than 150 mg/kg) causes hepatotoxicity by saturating the sulfation pathway and accumulation of toxic metabolites like N-acetyl-p-benzoquinone (NAPQI) via cytochrome P450s, resulting in decreased hepatocellular glutathione (GSH) levels and generating an imbalance between the production and removal of free radicals (Lancaster et al., 2015 ▶; Lee, 2017 ▶). Subsequently, if GSH is not replenished, excess NAPQI covalently binds to hepatocyte proteins and DNA, triggering oxidative stress damage, and increased lipid peroxidation and induced production of reactive oxygen species (ROS) and inflammatory mediators such as nitric oxide (NO) and cytokines (Bradley et al., 1982 ▶; McGill et al., 2012 ▶; Song et al., 2014 ▶; Xie et al., 2015 ▶). In addition, there is also extravasation of serum aminotransferases, ALT and AST, and hepatobiliary injury biomarkers (Alkaline phosphatase (ALP), and Gamma-glutamyl transferase (γ-GT)), indicating loss of functional integrity of the liver cell (Eesha et al., 2011 ▶; Lee, 2017 ▶; Robles-Diaz et al., 2015 ▶), in addition to causing severe hepatotoxicity and hepatocellular necrosis (McGill et al., 2012 ▶; Xie et al., 2015 ▶). Thus, any compound that can inhibit these cascade bindings may have positive effects on APAP-induced liver injury. Within this context, we chose to study β-myrcene (MYR), a very promising monoterpene, found in several plant extracts such as lemon balm, rosemary, lemongrass, among others. It has several pharmacological properties, such as antioxidant and anti-inflammatory, analgesic, sedative, antimicrobial and antinociceptive effects (Behr and Johnen, 2009 ▶; Ciftci et al., 2011 ▶; Hoseini et al., 2019 ▶; Paula-Freire et al., 2015 ▶; Rufino et al., 2015 ▶). However, the hepatoprotective effect of MYR in APAP-induced liver damage has not yet been discovered. Therefore, in the present work, the protective effects of MYR on liver damage induced by paracetamol were investigated to develop an effective hepatoprotective agent to protect against oxidative hepatocellular damage.

## Materials and Methods

**Chemicals**

All chemicals, including β-myrcene, were purchased from Sigma-Aldrich (St. Louis, MO, USA) unless otherwise stated. Standard commercial silymarin (SLM) and acetaminophen (APAP) were purchased from a public pharmacy and administered as a solution with carboxymethylcellulose (CMC). Kits for the analysis of ALT, AST, ALP, and γ-GT were obtained from Gold Analisa Diagnosticar, Brazil. All materials used were of analytical purity.

**Animals**

Experiments were executed on male Balb/c mice (6 weeks old), weighing 25±2 g, provided by the Central Animal House of the State University of Maringá, PR, Brazil. The mice were kept in our animal house facilities in standard laboratory conditions at a constant temperature of 23±2°C and 60% humidity, in a 12 hr light-dark cycle. The animals were acclimated under these conditions for three days before starting the study. They had unrestricted access to standard pellet food and water. The study protocol was revised and approved by the Committee on Animal Care of the State University of Maringá with the ethical number: CEUA/UEM Nº 2534270418 and conducted according to the international guidelines for the use and care of laboratory animals.

**Experimental design**

A total of 40 Balb/c mice were randomly divided into five groups as follows: 1) Normal control group that received only carboxymethylcellulose (CMC), the vehicle used to dissolve APAP and MYR; 2) APAP group that received a single dose of APAP (250 mg/kg) orally on day 7; 3) Silymarin group in which, animals received 200 mg/kg/day of silymarin, which is recognized and used as a reference drug for its hepatoprotective properties (Elsayed Elgarawany et al., 2020 ▶; Papackova et al., 2018 ▶); and 4 and 5) Pre-treatment groups in which, mice were treated with 100 or 200 mg/kg/day of MYR. After seven consecutive days, except for the normal control group, animals received a single APAP dose (250 mg/kg). Twelve hours after administration of APAP, the animals were anesthetized with 3% isoflurane by inhalation and the blood sample was collected from the cave vein in heparin-containing tubes for biochemical analyses as described by previous works (Cardia et al., 2021 ▶; Grespan et al., 2014 ▶; Uchida et al., 2017 ▶). Next, the livers were immediately and carefully removed, and one part was considered for determination of levels of hepatic tissue markers myeloperoxidase (MPO) activity and nitric oxide (NO), and another was clamped in nitrogen and stored at −80°C for oxidative status measurements. The other parts of liver tissue were kept in formalin 10% for histopathological examinations.

**Serum enzymes analysis**

The blood samples collected from animals were centrifuged at 5,000 g for 15 min at 4°C for biochemical assessment. The activity level of liver enzymes, including ALT, AST, ALP, and γ-GT, was selected to estimate some biochemical parameters functionality and cellular damage of liver tissue using the corresponding commercial kits according to the manufacturers.

**Myeloperoxidase activity assay**

The liver samples were homogenized in ten volumes of sodium phosphate buffer in a Potter homogenizer on ice and centrifuged at 5000 g for 15 min at 4°C. An aliquot of the supernatant (10 μl) was transferred in triplicate to each well of the microplate. Then, 200 μl of solution that contained O-dianisidine (16.7 mg), distilled water (90 ml), potassium phosphate buffer (10 ml), and 1% hydrogen peroxide (50 μl), was added. The enzymatic reaction was stopped by addition of sodium acetate in each well. Colorimetric measurements determined MPO activity in the liver in an ELISA plate reader (Asys Expert Plus) at the wavelength of 460 nm (Bradley et al., 1982 ▶). 

**Measurement of nitric oxide production**

The nitrite concentration was determined using the Griess method to estimate NO production (Cardia et al., 2018 ▶). Briefly, 50 μl of the supernatant from the liver tissue samples was plated in triplicate into a 96‐well plate and the same volume of Griess reagent (0.1% naphthyl ethylenediamine and 1% sulfanilamide in 5% H_3_PO_4_ solution) added to each well and incubated for 10 min at room temperature. The nitrite concentration in the liver tissue samples was measured at 550 nm using an ELISA plate reader and derived from the precalibrated standard NaNO_2_ curve.

**Determination of oxidative stress markers**

Malondialdehyde (MDA) is a degraded oxidative lipid product from cell membranes and is an indicator of oxidative stress. This study measured MDA using the TBARS (thiobarbituric acid-reactive substances) assay (Buege and Aust, 1978 ▶). The levels of TBARS were measured at 532 nm using a standard curve prepared with 1,1′,3,3′-tetraethoxypropane, and values are expressed as nmol (mg protein)^ −1^. Also, ROS levels were determined in liver homogenate supernatants using 2′,7′‐dichlorofluorescein diacetate (DCFH‐DA) assay, which quantifies the oxidation of DCFH-DA to the fluorescent 2′,7′-dichlorofluorescein (DCF) in the presence of ROS (Rodrigues Siqueira et al., 2005 ▶). Briefly, the formation of DCF was measured by spectrofluorimetry (Shimadzu RF-5301, 504 nm for excitation and 529 nm for emission). The results are expressed as nmol/mg protein using a standard curve prepared for DCF. Antioxidant enzymes activities were assayed by spectrophotometry in the supernatants of the liver homogenate. Catalase (CAT) activity measurement is based on determining the decomposition rate of the H_2_O_2_ substrate, measuring the change in absorbance at 240 nm. Superoxide dismutase (SOD) activity was estimated according to the pyrogallol autoxidation method measured at 420 nm (Marklund and Marklund, 1974). Reduced (GSH) and oxidized (GSSG) glutathione were determined in liver homogenate spectrofluorimetrically (excitation 350 nm and emission 420 nm) using the o-phthalaldehyde (OPT) assay (J.Hissin and Hilf, 1976 ▶). GSH and GSSG were calculated using a standard curve, and values are reported in nmol/mg protein units. 

**Histopathological analysis**

Liver samples of each animal were collected and preserved in formalin 10% solution, dehydrated in graduate ethanol, and embedded in paraffin. Embedded liver tissues were cut into sections of 5-μm thickness using a microtome then, mounted on glass slides. Slides were stained with hematoxylin and eosin (H&E) and examined microscopically for structural changes to evaluate liver damage. 

**Statistical analysis**

All data were analyzed and are reported as the mean±standard error of the mean. Tukey's test analyzed the significance among the different groups following one-way analysis of variance (ANOVA). GraphPad Prism Version 5.0 software (Graph Pad Software Inc., San Diego, CA, USA) was used for all statistical analyses, and differences were considered significant at p≤0.05.

## Results

**Effect of MYR on serum biochemical markers**

**Figure 1:**
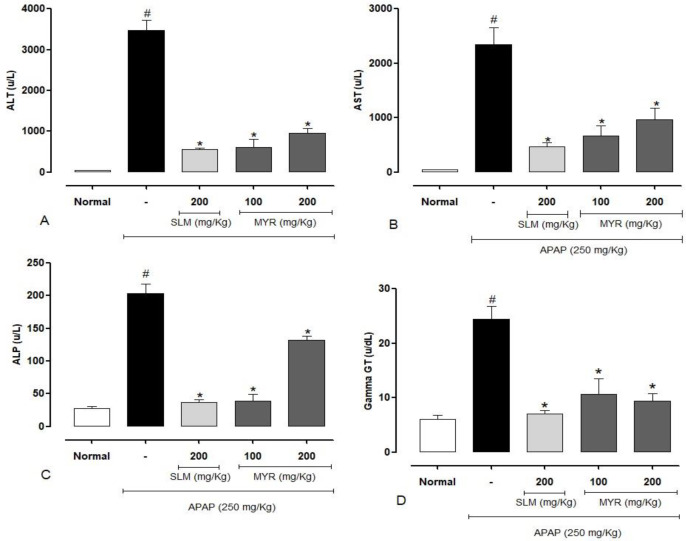
Effect of β-myrcene (MYR) and Silymarin (SLM) on alanine aminotransferase (A), aspartate aminotransferase (B), alkaline phosphatase (C) and gamma-glutamyl transferase (D). Results are expressed as mean±SEM, n=8/group. #(p<0.01) statistically significant compared to the normal animals. *(p<0.01) statistically significant compared to the APAP-treated animals

After the dose of APAP (250 mg/kg), the mice showed severe liver damage, as indicated by increases in serum liver enzymes (ALT, AST, ALP, and γ-GT) (Figure 1). We found that pretreatment with the two doses of MYR (100 mg/kg and 200 mg/kg) significantly prevented changes in ALT by 82.4 and 72.3%, respectively, AST by 71.4 and 59.1%, respectively, ALP by 80.9 and 35.0%, respectively and γ-GT by 55.7 and 61.4%, respectively, when compared to the APAP group (p<0.01) and a similar effect was observed for silymarin (reference drug) treatment (p<0.01). 

**MYR decreased MPO activity and NO production**

**Figure 2:**
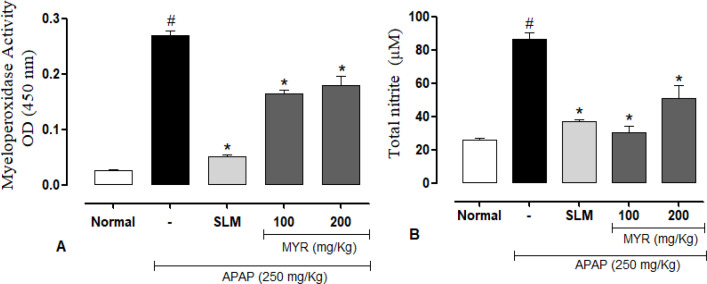
Effect of β-myrcene (MYR) and Silymarin (SLM) on (A) myeloperoxidase activity and (B) nitric oxide production. Results are expressed as mean±SEM. n=8/group. ^#^p<0.001 versus the control group. *p<0.001 MYR- or SLM-pretreated groups versus the APAP group

As shown in Figure 2A, the levels of hepatic MPO were markedly increased after APAP injection. On the other hand, pre-treatment with MYR at doses 100 and 200 mg/kg significantly decreased hepatic MPO content to about 37% and 30%. Furthermore, the NO production was also tested, and results are shown in Figure 2B. A single dose of APAP significantly elevated NO production in the liver when compared with the control animals. However, MYR treatment (100 and 200 mg/kg) significantly decreased the NO production compared with the APAP group in by 64 and 41%. The SLM group showed significantly decreased MPO activity and NO concentration (p<0.001) in the liver compared to the APAP-group.

**Determination of oxidative stress markers**

**Figure 3:**
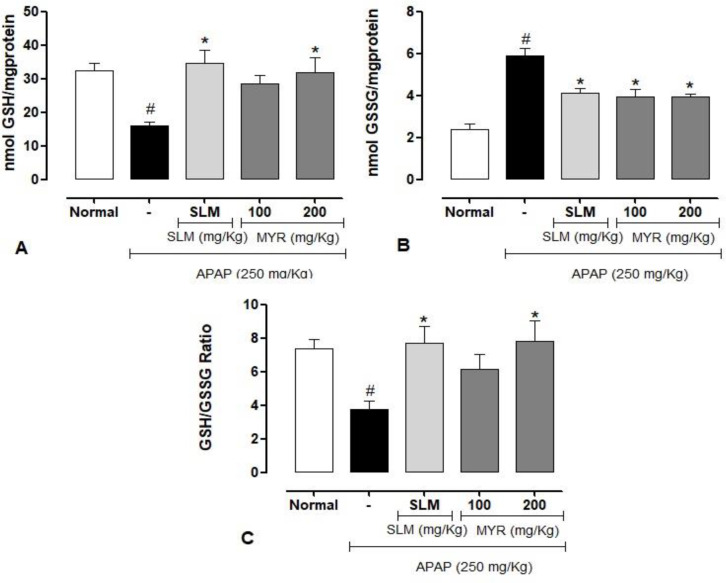
GSG and GSSH content in the liver. (A) GSG, (B) GSH and (C) GSH/GSSG. Data are presented as the mean±SEM, n=8/group. #(p<0.001) statistically significant compared to the normal animals. *p<0.001 statistically significant compared to the APAP group

In the experiment, we evaluated whether pretreatment with MYR could protect against hepatotoxicity by inhibiting oxidative stress. Levels of different oxidative stress markers and activities of antioxidant enzymes in the liver were determined. As shown in Figure 3, a toxic dose of APAP significantly reduced the levels of GSH and the ratio of GSH/GSSG while increasing the levels of GSSG compared with the normal animals (p<0.001). 

However, pretreatment with MYR 100 and 200 mg/kg significantly restored the APAP-induced GSH depletion. In addition, MYR 100 and 200 mg/kg reduced the increase of the liver GSSG content by 33 and 32%, respectively. The pretreatment with MYR 200 mg/kg increased the ratio of GSH to GSSG to levels comparable to those of the normal animals (p<0.01). A similar effect was observed for SLM (reference drug). Table 1 shows significantly increased levels of protein carbonyl, reactive oxygen species (ROS), and lipid peroxidation (TBARS) in the APAP-treated mice when compared with the control group (p<0.001), indicating that APAP induced oxidative stress in the liver. In contrast, pretreatment with MYR (200 mg/kg) effectively diminished the levels of ROS and MDA compared with the APAP group (p<0.001). The levels were similar to those of the SLM group. 

To further confirm the antioxidant effects of MYR, the activities of enzymes that protect against oxidative stress were measured. It was found that 12 hr after APAP-induced liver injury, the activities of hepatic SOD and CAT decreased significantly (p<0.001) compared to the normal animals (Table 1). However, these changes were attenuated in animals pretreated with MYR 100 and 200 mg/kg, inducing a significant increase in CAT by 86 and 85%, respectively and SOD by 107 and 135%, respectively compared with those of the APAP group. 

**Histopathological evaluation**

**Figure 4:**
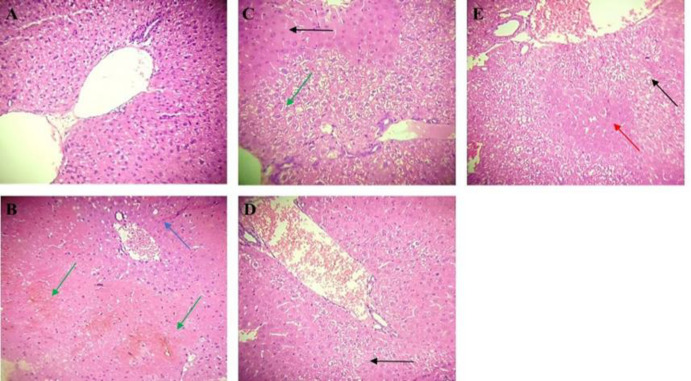
Results are expressed as mean±S.E.M. n=8/group Histopathological examination of liver tissue (H&E) (10X). Liver sections of the normal animals (A). APAP group (B). Silymarin control at 200 mg/kg + APAP (C). In, groups D and E, MYR 100 and 200 mg/kg + APAP, respectively, give a preventive effect against APAP-stimulated liver dysfunction. Areas of degenerated hepatocytes are pointed out by black arrow, and preserved hepatocytes are pointed out by red arrow. Loss of the normal architecture of the liver parenchyma, dilatation of the centrilobular vein and sinusoid congestion and hemorrhage (blue arrow). Areas of hydropic degeneration and necrosis of hepatocytes (green arrow)

Histopathological examination of hepatic sections in the APAP group showed severe changes characterized by loss of tissue architecture, centrilobular vein dilatation, sinusoid congestion, and hemorrhage, and areas of hydropic degeneration and zonal hepatocyte necrosis. In contrast, liver segments had well-defined structures and arrangements of hepatic cells with conserved cytoplasm and the central vein (Figure 4). Group treated with silymarin showed hepatocytes with pyknotic nuclei. However, APAP-induced hepatic necrosis was ameliorated by pre-administration of MYR (100 and 200 mg/kg). The treatment with MYR at a dose of 100 mg/kg showed areas of mildly degenerated hepatocytes. Furthermore, treatment with MYR 200 mg/kg showed areas of vacuolar degeneration and hepatocytes presenting standard architecture, compared to the APAP-administered group. We can confirm that MYR treatment can prevent liver tissue damage induced by APAP. 

**Table 1:** Effects of the pretreatment with MYR on liver oxidative stress parameters

	Normal	APAP	SLM	MYR 100 mg/kg	MYR 200 mg/kg
TBARS, nmol/MDA/mgprt	0.79±0.01	1.40±0.1^#^	0.95±0.06^*^	0.96±0.02^*^	0.95±0.09^*^
ROS, nmol/mg	0.74±0.07	1.58±0.1^#^	0.80±0.05^*^	1.25±0.1	1.17±0.06^*^
CAT, mmol/min·mgprt	1038±90	253±28.5^#^	467±25.8^*^	472±34.3^*^	470±19.6^*^
SOD, U/mgprt	2.88±0.1	1.40±0.1^#^	2.28±0.06	2.90±0.18^*^	3.03±0.2^*^

Results are expressed as mean±S.E.M. n=8/group #p<0.001 statistically significant compared to the normal animals. *p<0.001 statistically significant compared to the APAP-treated animals. MYR: β-myrcene. APAP: N-acetyl-p-aminophenol, SLM: Silymarin, TBARS: Thiobarbituric acid-reactive substances, ROS: reactive oxygen species, CAT: Catalase, SOD: Superoxide dismutase, and U·mg^−1^: Units per mg protein.

## Discussion

We report, for the first time, that MYR relieves ALF through anti-inflammatory, antioxidant, and anti-apoptotic effects. In the present study, APAP highly increased serum AST, ALT, ALP, and γ-GT. These enzymes are released into the blood after hepatocytes injury, and their elevated levels 

in the circulation indicate loss of functional integrity of hepatic cells (Eesha et al., 2011 ▶; Lee, 2017 ▶; Robles-Diaz et al., 2015 ▶). However, we found that pretreatment with MYR can prevent APAP-induced hepatic damage because it decreased the liver enzymes at both doses (Figure 1). A similar effect was seen with SLM treatment. Silymarin is recognized and used as a reference drug for its several hepatoprotective properties already reported (Elsayed Elgarawany et al., 2020 ▶; Papackova et al., 2018 ▶). Uchida et al. (Nancy Sayuri Uchida, Silva-Filho, Aguiar, et al., 2017) also obtained similar results using the essential oil of *Cymbopogon citratus* in an experimental model of liver injury induced by paracetamol, in which beta-myrcene was the main constituent of the oil. 

These changes were further confirmed by histopathological analysis of liver tissue. The groups pretreated with MYR showed an improvement in liver damage with the normal architecture of hepatocytes, reduction of hyperemia, infiltration of inflammatory cells, necrosis, and vacuolar degeneration (Figure 4), similar to the SLM-treated group, suggesting that MYR could restore hepatic cell function and integrity, therefore decreasing the leakage of the serum aminotransferases into the blood. Current experimental evidence demonstrates that oxidative stress is an essential factor in liver dysfunction in mice treated by APAP (Jaeschke et al., 2012 ▶). Thus, to confirm the hepatoprotective effect of MYR, we determined some parameters related to oxidative stress, including GSH, ROS, and MDA. 

GSH is a powerful antioxidant and can react with NAPQ to decrease toxic responses and protect cells from oxidative damage. Intense exposure to xenobiotics decreases hepatic GSH concentrations (Bayrak et al., 2008 ▶; Yan et al., 2018 ▶). In addition, the hepatic content of MDA is used as a biomarker of oxidative damage to assess lipid peroxidation (Agarwal et al., 2011 ▶). Our study demonstrates that APAP overdose caused a reduction in GSH levels and increased ROS and MDA content, suggesting that APAP induced a redox imbalance to accumulate ROS, lipid peroxidation, and formation of protein oxidation products. Furthermore, our study found that the MYR pretreatment reversed these parameters to levels of the control group. 

Several studies investigated the antioxidant effect of MYR in different experimental models and reported similar findings with our study. A study demonstrated that MYR treatment reduced renal inflammation and oxidative stress by decreasing lipid peroxidation and significantly increasing glutathione levels compared to untreated animals (Islam et al., 2020 ▶). Furthermore, it was also shown that MYR treatment caused a significant decrease in TBARS levels and an increase in GSH levels and SOD and CAT activities in the cardiac tissue of mice after ischemia/reperfusion (Burcu et al., 2016 ▶). These experimental results suggest that the MYR hepatoprotective activity might be associated with its antioxidant capacity.

We evaluated the main free-radical scavenging enzymes to confirm the hepatoprotective effect of MYR associated with oxidative stress. SOD and CAT are essential enzymes in the enzymatic antioxidant defense system, and reductions in their activities can result in several deleterious effects (Arauz et al., n.d.; Younus, n.d.). In the present work, oral administration of a toxic dose of APAP caused a significant decrease in serum SOD and CAT levels. However, SOD and CAT activities increased in MYR-treated mice. Furthermore, the increased activity of SOD and CAT reduced NAPQI and ROS, promoting attenuation of liver damage.

These results meet the agreement of other studies where treatment with MYR has been shown to exert protective effects on kidney damage due, in part, to its antioxidant properties, protecting against depletion of CAT and SOD activities (Islam et al., 2020 ▶). In addition, MYR has also been shown to increase GSH and the SOD and CAT activities, protecting the liver from oxidative damage induced by 2,3,7,8-tetrachlorodibenzo-p-dioxin (Ciftci et al., 2011 ▶). Other studies also report that MYR and other monoterpenes can protect human cells against oxidative damage (Mitić-Ćulafić et al., 2009 ▶; Wang et al., 2005 ▶). Thus, we can suggest that MYR treatment can reduce reactive free radicals, thus, attenuating oxidative damage to liver tissue and improving the activity of antioxidant enzymes. 

Many studies have shown that APAP-induced hepatotoxicity can be attenuated by decreasing the inflammatory response (Cardia et al., 2021 ▶; da Rocha et al., 2017 ▶; Freitag et al., 2015 ▶; N.S. Uchida et al., 2017). It has been demonstrated that APAP overdose causes an increase in NO formation. NO reacts rapidly with ROS to form peroxynitrite, an oxidant that causes several deleterious effects on neutrophils, aggravating lipid peroxidation (Saito et al., 2010 ▶; Song et al., 2014 ▶). Furthermore, some studies have shown that MPO activity is directly proportional to the degree of neutrophil infiltration. Exacerbated migration of these cells can aggravate hepatotoxicity (Bradley et al., 1982 ▶; Mullane et al., 1985 ▶). In this way, we determined the activity of MPO as an indirect marker of inflammation. In the present study, pretreatment with MYR was able to reduce increased levels of NO and MPO. Additionally, pro-inflammatory cytokines such as interleukin-1β (IL-1β), interleukin-6 (IL-6), and tumor necrosis factor-α (TNF-α) are involved in APAP-induced hepatotoxicity (Hinson et al., 2010 ▶). In this sense, other studies reported that MYR could act by reducing several inflammatory mediators. A study determined that MYR can reduce pro-inflammatory cytokines (Islam et al., 2020 ▶). Furthermore, it was also shown that MYR inhibited lipopolysaccharide-stimulated NO production in macrophages and reduced the expression of NF-κB and inducible nitric oxide synthase (iNOS) (Doan et al., 2021 ▶). Thus, our results suggest that MYR can prevent hepatotoxicity, in part, by attenuating the inflammatory response induced by APAP.

In summary, our study is the first to deal with the MYR protective effect against APAP-induced liver damage. We provide evidence that MYR administration significantly inhibited APAP-induced liver damage. Its hepatoprotective effect can be mediated by the normalization of several biochemical characteristics of liver function and oxidative stress and improving histopathological parameters due to its antioxidant activity. Thus, our study proposes that MYR could be a viable therapeutic strategy for APAP-induced acute liver injury and other oxidative stress-mediated toxicities.

## Conflicts of interest

The authors have declared that there is no conflict of interest.
